# ^125^Iodine monotherapy for Japanese men with low- and intermediate-risk prostate cancer: outcomes after 5 years of follow-up

**DOI:** 10.1093/jrr/rrt113

**Published:** 2013-10-08

**Authors:** Akane Sekiguchi, Hiromichi Ishiyama, Takefumi Satoh, Kenichi Tabata, Shouko Komori, Hideyasu Tsumura, Shogo Kawakami, Itaru Soda, Masatsugu Iwamura, Kazushige Hayakawa

**Affiliations:** 1 Department of Radiology and Radiation Oncology, Kitasato University School of Medicine, 1-15-1 Kitasato, Sagamihara, Japan; 2 Department of Urology, Kitasato University School of Medicine, 1-15-1 Kitasato, Sagamihara, Japan

**Keywords:** prostate cancer, brachytherapy, low dose rate, ^125^I, monotherapy

## Abstract

Data from 305 Japanese men with low-risk (*n* = 175) or intermediate-risk (*n* = 130) prostate cancer who underwent ^125^I monotherapy were retrospectively analyzed. Of the 305 patients, 93 received hormonal therapy for a median of 6 months (range, 1–33 months) before implantation. The prescribed dose to the prostate plus 3- to 5-mm margin was set at 145 Gy. The mean dose to 90% of the prostate volume at 1 month (D90) and the prostate volume receiving at least 100% dose at 1 month (V100) were 173.4 Gy and 95.8%, respectively. The median follow-up was 66 months (range, 12–94 months). The 5-year biochemical non-evidence of disease rate was 95.5% (low-risk, 94.2%; intermediate-risk, 97.3%). The 5-year freedom from clinical failure rate was 98.9% (low-risk, 98.9%; intermediate-risk, 99.2%).The initial prostate-specific antigen level was identified as a significant predictive factor for biochemical recurrence (*P* = 0.029). The late Grade 3 genitourinary toxicity rate was 2.0%. No patients displayed late gastrointestinal toxicity of Grade 3 or worse. Monotherapy with ^125^I showed excellent outcomes with limited morbidity for Japanese men with low- and intermediate-risk prostate cancer after 5 years of follow-up.

## INTRODUCTION

Permanent interstitial implantation of ^125^I for prostate cancer is now a popular treatment option for localized prostate cancer in Japan, where almost a decade has passed since the introduction of this treatment. Although several useful reports from Japanese institutions have described toxicity, dosimetry and technical problems, long-term clinical results of this treatment have been lacking. We have treated low-risk patients and some intermediate-risk patients with ^125^I monotherapy from the start of this treatment, and over 1000 patients have now been treated.

The purpose of this study was to report our results for 300 prostate cancer patients treated using ^125^I monotherapy after 5 years of follow-up.

## MATERIALS AND METHODS

### Patients

The institutional review board approved this retrospective study. A total of 306 consecutive Japanese men with low- and intermediate-risk prostate cancer underwent ^125^I monotherapy between May 2004 and August 2007. Of these 306 patients, 1 patient died of cerebral apoplexy before the first follow-up examination [1]. We therefore retrospectively analyzed data from 305 of the 306 patients. Patient characteristics are shown in Table 1. Patients with clinical Stage T1c or T2a who also had a prostate-specific antigen (PSA) level ≤10 ng/ml and a biopsy Gleason score (GS) of 2–6 were defined as a low-risk group. Conversely, patients with clinical Stage T2c or a PSA level >20 ng/ml or a biopsy GS of ≥8 were defined as a high-risk group. The remaining patients were defined as an intermediate-risk group. In our treatment protocol, intermediate-risk patients with low PSA level ( ≤ 15 ng/ml) were basically treated using^125^I monotherapy.

**Table 1.** Patient characteristics

*n*		305	
Age (years)		68	(51–84)
Initial PSA (ng/ml)		6.22	(1.48–19.3)
Gleason Score			
	≤6	178	
	3 + 4	90	
	4 + 3	37	
T stage			
	T1c	251	
	T2a	47	
	T2b	7	
Risk group			
	Low	175	
	Intermediate	130	
Neoadjuvant hormone			
yes		93	
no		212	

Values represent median (range) or number. GnRHa = Gonadotropin-releasing hormone agonist, MAB = maximum androgen blockade, PSA = prostate-specific antigen.

Clinical staging was decided from the results of digital rectal examinations and bone scintigraphy. Computed tomography (CT) and/or magnetic resonance imaging (MRI) were also used to determine the T stage.

Basically, 3 months of hormonal therapy was performed in patients with a large prostate gland (≥40 cm^3^) before implantation. Some patients, however, received hormonal therapy before admission to our hospital.

### Treatment

The procedure was performed in the extended lithotomy position under observation using transrectal ultrasonography (US). Seeds were placed one by one transperineally through needles attached to a Mick applicator (Mick Radio-Nuclear Instrument, Mount Vernon, NY). In the first 27 patients (treated from May 2004 to October 2004), dosimetry was planned based on US performed 4 weeks before implantation. In the next 86 patients (treated from October 2004 to October 2005), dosimetry was planned intraoperatively, based on US performed just before implantation in the anesthetized patient. In the remaining 192 patients (treated from October 2005 to August 2007), an interactive plan technique [2] was used. All patients were hospitalized the day before seed implantation, and discharged 2 d after implantation. Almost all dosimetry was planned using Interplant 3.2 software (CMS, St Louis, MO). The prescribed dose to the prostate plus a 3- to 5-mm margin was set at 145 Gy.

### Follow-up

Patients were monitored by measuring serum PSA levels every 3 months for the first year, and every 3–6 months thereafter. Biochemical failure was defined according to the Phoenix definition [3]. Urinary and rectal morbidity were assessed using the Radiation Therapy Oncology Group (RTOG) scale [4] and National Cancer Institute Common Toxicity Criteria (NCI-CTC) version 3. All patients received α-blockers just after seed implantation to relieve urinary symptoms.

### Dosimetric analysis

Post-implantation dosimetric analysis was performed using CT scans performed at 1 d and 30 d after implantation according to the recommendations of the American Brachytherapy Society [5]. Dose–volume histograms (DVHs) were calculated for every patient.

The urethra was contoured on the same slices as the prostate contouring with a balloon catheter on the Day 1 CT. On the Day 30 CT, however, the surrogate urethra assumed to be at the geometric center of the prostate was contoured on the same slices as the prostate. The rectal wall was contoured, including the sphincter muscle, on the same slices as the prostate contouring.

### Statistics

Univariate analysis (log-rank) was used to examine the predictive value including hormonal therapy, risk criteria, age, GS, prostate volume, clinical T stage, number of patients experienced at the time of treatment, dose to 90% of prostate volume at 1 month (D90), number of seeds, number of needles, initial PSA, PSA doubling time, body mass index, and testosterone value. Multivariate analysis was performed using Cox regression analysis. Statistical analysis for genitourinary (GU) and gastrointestinal (GI) toxicities was not performed in this study.

## RESULTS

**Fig. 1.**
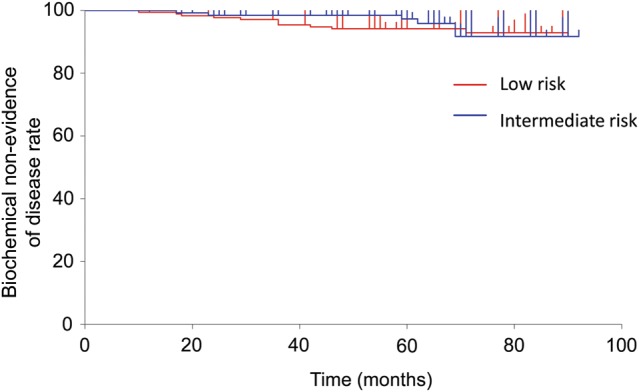
Biochemical non-evidence of disease rate.

The median follow-up was 66 months (range, 12–94 months). The 5-year biochemical non-evidence of disease rate (bNED) was 95.5% (low-risk group, 94.2%; intermediate-risk group, 97.3%; Fig. 1). The 5-year freedom from clinical failure rate was 98.9% (low-risk group, 98.8%; intermediate-risk group, 99.2%). Two patients died of lung cancer, and the 5-year overall survival rate (OS) was 99.6%.

Biochemical failure according to the Phoenix definition occurred in 17 patients. Of those 17 patients, 5 patients showed PSA reduction after temporary rises (PSA bounce), 2 patients revealed lymph-node recurrence on CT, and 2 patients started hormonal therapy. Of the remaining 8 patients, 7 patients underwent prostate biopsy. Evidence of viable cells was found in only 1 patient.

Details of DVH parameters are shown in Table 2. Urinary and rectal morbidity as assessed using RTOG and NCI-CTC scales are shown in Tables 3 and 4, respectively. Acute morbidity was defined as morbidity occurring ≤12 months after implantation, while late morbidity was defined as morbidity occurring >12 months, according to RTOG criteria.

**Table 2.** Treatment-related factors and postimplant dosimetric factors

Factors		Mean	SD
Treatment-related factors			
	Number of seeds	91	(15.8)
	Number of needles	24.1	(5.6)
	Preimplant US prostate volume (ml)	31.4	(8.8)
Postimplant dosimetric factors			
	pD90 (Gy)	173.4	(25.4)
	pV100 (%)	95.8	(5.9)
	pV150 (%)	64.7	(15.5)
	uD90 (Gy)	157.2	(30.2)
	uD10 (Gy)	226.1	(33.5)
	uV200 (ml)	0.0	(0.0)
	rV100 (ml)	0.9	(0.8)
	rV150 (ml)	0.1	(0.2)

pD90 = dose to 90% of prostate volume at 1 month, pV100 = prostate volume receiving at least 100% dose at 1 month, pV150 = prostate volume receiving at least 150% dose at 1 month, uD90 = dose to 90% of urethral volume at 1 month, uD10 = dose to 10% of urethral volume at 1 month, uV200 = urethral volume receiving at least 200% dose at 1 month, rV100 = rectal volume receiving at least 100% dose at 1 month, rV150 = rectal volume receiving at least 150% dose at 1 month, SD = standard deviation.

**Table 3.** Acute and late genitourinary and gastrointestinal morbidity with respect to RTOG criteria

	Grade							
	0		1		2		3	
Acute (within 1 year)								
GU	34	(11.1%)	194	(63.6%)	63	(20.7%)	14	(4.6%)
GI	247	(81.0%)	54	(17.7%)	4	(1.3%)	0	(0.0%)
Late (after 1 year)								
GU	104	(34.1%)	174	(57.0%)	21	(6.9%)	6	(2.0%)
GI	231	(75.7%)	70	(23.0%)	3	(1.0%)	0	(0.0%)

GU = genitorurinary toxicity, GI = gastrointestinal toxicity

**Table 4.** Urinary and rectal morbidity with respect to NCI-CTC ver.3

	Grade							
	0		1		2		3	
Frequency	52	(17.0%)	173	(56.7%)	74	(24.3%)	6	(2.0%)
Retension	84	(27.5%)	197	(64.6%)	13	(4.3%)	11	(3.6%)
Miction pain	164	(53.8%)	130	(42.6%)	11	(3.6%)	0	(0.0%)
Incontinence	291	(95.4%)	8	(2.6%)	6	(2.0%)	0	(0.0%)
Hematuria	255	(83.6%)	44	(14.4%)	6	(2.0%)	0	(0.0%)
Proctitis	255	(83.6%)	48	(15.7%)	2	(0.7%)	0	(0.0%)
Incontinence (anal)	303	(99.3%)	2	(0.7%)	0	(0.0%)	0	(0.0%)
Diarrhea	292	(95.7%)	12	(3.9%)	1	(0.3%)	0	(0.0%)
Rectal bleeding	232	(76.1%)	69	(22.6%)	4	(1.3%)	0	(0.0%)

Diarrhea grade was counted by the number of bowel movements and did not necessarily mean that the patient had loose stools, with few patients experiencing loose or watery stools. Proctitis and hemorrhage might include not only the effects of radiation, but also hemorrhoids, due to the difficulties of differential diagnosis.

GU symptoms such as frequency, micturition pain and retention were common, but usually not severe. The late Grade 3 GU toxicity rate was only 2.0%. GI symptoms such as proctitis and rectal bleeding were common, but very mild.

Table 5 shows the results of uni- and multivariate analyses of bNED. Only initial PSA level was detected as a significant predictor of biochemical recurrence on univariate analysis.

**Table 5.** Univariate and multivariate analysis of biochemical non-evidence of disease rate

		Univariate		Multivariate	
Variant		*P*-value	HR	*P*-value	HR
Hormonal therapy	No vs Yes	0.132	2.953		
Damico risk criteria	Low vs Intermediate	0.429	1.526		
Age	≤70 vs >70	0.931	0.958		
Gleason score	<3 + 4 vs >4 + 3	0.071	0.368	0.206	2.065
Prostate volume (ml)	≤32 vs >32	0.617	1.285		
T stage	1c vs ≥2a	0.251	3.072		
Experience (patients)	<152 vs ≥153	0.267	1.744		
D90 (Gy)	≤145 vs >145	0.158	2.195		
Initial PSA (ng/ml)	<6 vs ≥6	**0.029**	0.310	0.062	2.912
Doubling time (years)	≥2 vs <2	0.890	0.921		
(hormone naïve only)					
	≥1 vs <1	0.489	1.448		
Testosterone	≥6 vs <6	0.686	1.327		
(hormone naïve only)					
BMI	≤25 vs >25	0.482	0.697		

PSA = prostate specific antigen, HR = hazard ratio, D90 = dose to 90% of prostate volume at 1 month.

## DISCUSSION

Our results suggest that ^125^I monotherapy offers an effective treatment, not only for low-risk prostate cancer, but also for intermediate-risk prostate cancer. Evidence of local recurrence confirmed by biopsy was seen in only one patient. Because relatively high D90 had been prescribed to our patients, favorable local control might have been achieved even in intermediate-risk patients.

Well-known predictive factors such as GS, D90, and risk group were not detected as significant factors in this study. As few biochemical recurrences (17 of 305 patients) occurred in the follow-up period, the statistical power might have been insufficient to show significant differences. Initial PSA value was, however, detected as a significant predictor of biochemical recurrence, as described in other reports [6–8]. Although univariate analysis of initial PSA stratified by 6 ng/ml showed statistical significance (Table 5), an analysis of that stratified by 10 ng/ml did not show statistical significance.

Under the present protocol, no patients underwent external beam radiotherapy (EBRT). Controversy remains regarding the combination of EBRT with brachytherapy for intermediate-risk patients. The purposes of combining EBRT are: (i) covering extracapsular extension (ECE) with a sufficient dose; and (ii) increasing the biologically equivalent dose (BED). Regarding ECE coverage, Teh *et al*. [9] analyzed pathological features of 712 patients who underwent prostatectomy and reported that only 26% of patients exhibited ECE, with a median depth of 2.0 mm. Therefore, 87% of patients needed only a 2-mm margin to cover ECE. In addition, margin size can be changed depending on the risk of ECE by referring to useful information such as positive biopsy distribution or MRI. Our present monotherapy technique with 3- to 5-mm margins appears sufficient for ECE coverage.

Regarding BED, our dose (mean, 173 Gy) seems sufficient for low-risk prostate cancer and some intermediate-risk prostate cancer. This dose is equal to a BED of 183.4 Gy_2_ based on α/β = 2. This dose can be safely prescribed without EBRT. Although high-dose therapy (>220 Gy_2_) might be needed for high-risk and some intermediate-risk patients [10], dose escalation without EBRT carries a potential risk of severe toxicities. Because the dosimetries 1 month after implantation have significant deviations (Table 2), unintended low or high prescriptions inevitably occur. For example, our mean pD90 of 173 Gy has a standard deviation of 26 Gy. That means 32% of patients have a pD90 <147 Gy or >199 Gy. Meanwhile, EBRT can provide an intended prescription exclusive of geometric error. There is little risk of unintended low or high doses in EBRT. If a high dose >200 Gy is needed, addition of EBRT may offer a safe means of achieving dose escalation.

Uesugi *et al*. [11] reported Gleason Grade 4 as a significant prognostic factor associated with biochemical recurrence. Meanwhile, Stock *et al*. [12] reported that Gleason Grade 4 has no impact on biochemical failure. Although our univariate analysis also suggested the GS as close to a significant prognostic factor for biochemical recurrence (*P* = 0.07), multivariate analysis found no such tendency. Our relatively high prescribed dose might have diminished the impact of Gleason Grade 4. Based on our results, we have to emphasize that a treatment results of monotherapy in higher BED (>180 Gy_2_) and higher V100 (>95%), resulting in the excellent results seen for intermediate-risk patients and the lack of difference with low-risk patients.

Controversy remains regarding the use of hormonal therapy combined with brachytherapy for intermediate-risk patients. Merrick *et al*. [13] reported that hormonal therapy did not improve bNED for intermediate-risk patients. Similarly, our data did not show improvement in bNED for intermediate-risk patients (*P* = 0.500; data not shown). Meanwhile, Lee *et al*. reported that the 5-year bNED was improved with neoadjuvant hormonal therapy in intermediate-risk patients [14]. Interestingly, Henry *et al*. reported that the use of hormonal therapy was associated with ‘poorer’ bNED, particularly among intermediate-risk patients [15]. As some authors have suggested that hormonal therapy might be associated with risk of coronary artery disease [16], indications for hormonal therapy should be considered with caution. Thus, a randomized trial for intermediate-risk patients with ^125^I interstitial brachytherapy with and without neoadjuvant hormonal therapy is necessary. Further investigation is needed regarding good selection criteria for hormonal therapy.

In terms of toxicity, our previous report showed an acceptable toxicity rate after implantation [17], which has been confirmed with our updated results presented here. We plan to report on further analysis of relationships between radiation dose and toxicities in another article.

Although follow-up times have not been sufficient, favorable results have been reported from several other Japanese institutions [11,17,18]. Almost a decade after its introduction, it appears that ^125^I interstitial brachytherapy has successfully provided a safe and effective treatment for Japanese prostate cancer patients.

## CONCLUSION

In conclusion, monotherapy with ^125^I shows excellent outcomes with limited morbidity for Japanese men with low- and intermediate-risk prostate cancer after 5 years of follow-up.

## CONFLICT OF INTEREST

H.I., T.S. and K.H. received honoraria for lecture fees from Medicon Co. Ltd and Nihon Medi-Physics Co. Ltd.

## FUNDING

This work was supported by JSPS KAKENHI Grant Number 24791334.
